# Exploring the Archaeome: Detection of Archaeal Signatures in the Human Body

**DOI:** 10.3389/fmicb.2019.02796

**Published:** 2019-12-05

**Authors:** Manuela R. Pausan, Cintia Csorba, Georg Singer, Holger Till, Veronika Schöpf, Elisabeth Santigli, Barbara Klug, Christoph Högenauer, Marcus Blohs, Christine Moissl-Eichinger

**Affiliations:** ^1^Department of Internal Medicine, Medical University of Graz, Graz, Austria; ^2^Department of Pediatrics and Adolescent Surgery, Medical University of Graz, Graz, Austria; ^3^Institute of Psychology, University of Graz, Graz, Austria; ^4^BioTechMed-Graz, Graz, Austria; ^5^Department of Dental Medicine and Oral Health, Medical University Graz, Graz, Austria

**Keywords:** human archaeome, amplicon sequencing, human body, detection, methodology

## Abstract

Due to their fundamentally different biology, archaea are consistently overlooked in conventional microbiome surveys. Using amplicon sequencing, we evaluated methodological set-ups to detect archaea in samples from five different body sites: respiratory tract (nasal cavity), digestive tract (mouth, appendix, and stool) and skin. With optimized protocols, the detection of archaeal ribosomal sequence variants (RSVs) was increased from one (found in currently used, so-called “universal” approach) to 81 RSVs in a representative sample set. The results from this extensive primer-evaluation led to the identification of the primer pair combination 344f-1041R/519F-806R which performed superior for the analysis of the archaeome of gastrointestinal tract, oral cavity and skin. The proposed protocol might not only prove useful for analyzing the human archaeome in more detail but could also be used for other holobiont samples.

## Introduction

The importance of microbial communities to human and environmental health motivates microbiome research to uncover their diversity and function. While the era of metagenomics and metatranscriptomics has begun, 16S rRNA gene amplicon sequencing still remains one of the most used methods to explore microbial communities, mainly due to the relatively low cost, the number of available pipelines for data analysis, and the comparably low computational power required.

It has been recognized that methodological issues in sample processing can significantly influence the outcome of microbiome studies, affecting comparability between different studies (Clooney et al., 2016; de la Cuesta-Zuluaga and Escobar, 2016) or leading to an over-and/or under-estimation of certain microbial clades (Eloe-Fadrosh et al., 2016; Eisenstein, 2018). For better comparability among different studies, standard operational procedures for sampling, storing samples, DNA extraction, amplification and analysis were set-up [e.g., the Earth Microbiome Project (Gilbert et al., 2014) and the Human Microbiome Project (Methé et al., 2012)]. This includes the use of so-called “universal primers” (Caporaso et al., 2012; Klindworth et al., 2013; Walters et al., 2016), to maximally cover the prokaryotic diversity.

The human microbiome consists of bacteria, archaea, eukaryotes, and viruses. The overwhelming majority of microbiome studies is bacteria-centric, but in recent years, the number of human microbiome studies targeting eukaryotes (e.g., fungi), and viruses has increased (Seed, 2014; Zou et al., 2016; Halwachs et al., 2017). However, most microbiome studies still overlook the human archaeome (Eisenstein, 2018; Moissl-Eichinger et al., 2018). A few of the underlying reasons for the under-representation of archaea in microbiome studies are: (i) primer mismatches of the “universal primers” (Raymann et al., 2017); (ii) low abundance of the archaeal DNA in the studied samples (Mahnert et al., 2018); (iii) improper DNA extraction methods (Ghavami et al., 2015); and (iv) the incompleteness of the 16S rRNA gene reference databases due to missing isolates, especially for the DPANN superphylum (Castelle et al., 2015; Mahnert et al., 2018). Moreover, the clinical interest on archaea has been comparatively insignificant, as no archaeal pathogens have been identified (Gill et al., 2006).

Nevertheless, archaea (e.g., methanogens) are among the commensal microorganisms inhabiting the human body. Such archaea are regularly detected in the oral cavity and the gastrointestinal tract (Horz and Conrads, 2011; Gaci et al., 2014; Chaudhary et al., 2015; Nkamga et al., 2017). In the latter, they can even outnumber the most abundant bacterial species by as much as 14%, as revealed in a shotgun-based metagenomic analysis of gut samples of 96 healthy Russian adults (Tyakht et al., 2013).

Most studies of archaea in humans use either cultivation or qPCR-based detection methods (Dridi et al., 2009, 2012; Borrel et al., 2017; Grine et al., 2017; Koskinen et al., 2017; van de Pol et al., 2017; Wampach et al., 2017). Only a few 16S rRNA-based archaea-centric studies are available (Mihajlovski et al., 2010; Hoffmann et al., 2013; Koskinen et al., 2017; Moissl-Eichinger et al., 2017). These studies show that archaea are also present in the human respiratory tract (Koskinen et al., 2017) and on human skin in considerable amounts (Probst et al., 2013; Moissl-Eichinger et al., 2017).

It has also been shown that archaea reveal body site-specific patterns as do human-associated bacteria (Koskinen et al., 2017). For example, the gastrointestinal tract is dominated by methanogens, the skin by Thaumarchaeota, the lungs by Woesearchaeota, and the nasal archaeal communities are composed of a mixture of mainly methanogens and Thaumarchaeota. Together, these data demonstrate a substantial presence of archaea in some, or even all, human tissues.

As a logic progression from our previous studies, we have begun to optimize the detection of archaea commensals in humans. Specifically, we tested, *in silico* and experimentally, 27 different 16S rRNA gene-targeting primer pair combinations suitable for NGS amplicon sequencing with the goal of detecting the archaeal diversity in samples from different body sites, including: respiratory tract (i.e., nasal samples), digestive tract (i.e., oral biofilms, appendix biopsy specimens, and stool samples), and skin.

## Materials and Methods

### Selection of Samples and DNA Extraction

Representative samples from various body sites including the respiratory tract (nasal swabs), the digestive tract (oral biofilm, appendix biopsy, and stool samples) and skin swabs were selected for the comparison of amplification-based protocols.

Research involving human material was performed in accordance with the Declaration of Helsinki and was approved by the local ethics committee (Medical University of Graz, Graz, Austria).

Microbiome studies focused on bacteria have already been published using some of the samples used in this present study (Santigli et al., 2016; see Klymiuk et al., 2016; Koskinen et al., 2018). Details of the ethics approvals are provided in these prior studies for oral, nasal, and skin sample use.

Appendix and stool samples have been obtained covered by the ethics votes 25-469 ex12/13 and 27-151 ex 14/15 (informed consent was obtained from all participants or their parent/legal guardian).

Nasal swabs (*n* = 7) were obtained from healthy adult volunteers (18–40 years old) and were taken from the olfactory mucosa located at the ceiling of the nasal cavity using ultra minitip nylon flocked swabs (Copan, Brescia, Italy) (Koskinen et al., 2018). Oral samples (*n* = 7) were obtained using a standardized protocol for paper point sampling from healthy children (10 years old) who participated in a microbiome study investigating the subgingival biofilm formation (Santigli et al., 2016, 2017). Appendix samples (*n* = 6) were obtained during pediatric appendectomies from children (7–12 years old) with either acute or ulcerous appendicitis. Stool samples (*n* = 5) were obtained from healthy adult volunteers (18–40 years old) (Bagga et al., 2018), and from one patient (68 years old) with above average methane production after metronidazole treatment. Skin samples (*n* = 7) were obtained from healthy adult volunteers (18–40 years old) from either the back (*n* = 1) or the left forearm (*n* = 6) using BD Culture Swabs^TM^ (Franklin Lakes, NJ, United States).

Sample set 1 (one representative sample from each body site: nasal, oral, appendix, stool from patient with high methane production, and skin from the back) was used to initially evaluate the primers and methods, whereas sample set 2 (6 nasal samples, 6 oral samples, 5 appendices, 5 stool samples, and 7 skin samples) was then used for assessing the archaeal diversity, based on the chosen, optimal protocol.

In all cases, the genomic DNA was extracted by a combination of mechanical and enzymatic lysis. However, depending on the sample type, different protocols were used: for the stool samples approx. 200 mg of sample has been used for DNA extraction using the E.Z.N.A. stool DNA kit according to the manufacturer’s instruction. The DNA from the appendix samples was obtained using the AllPrep DNA/RNA/Protein Mini Kit (QIAGEN), before the DNA extraction, small pieces of cryotissue were homogenized 3 times for 30 s at 6500 rpm using the MagNALyzer^®^ instrument (Roche Molecular Systems) with buffer RTL and β-mercaptoethanol (according to the manufacturer’s instructions). For the nasal and skin samples from the forearm, the DNA was extracted using the FastDNA Spin Kit (MP Biomedicals, Germany) according to the provided instructions. The DNA from the oral samples and from the skin samples from the back were isolated using the MagnaPure LC DNA Isolation Kit III (Bacteria, Fungi; Roche, Mannheim, Germany) as described by Klymiuk et al. (2016) and Santigli et al. (2016).

### 16S rRNA Gene Primer Selection and Pre-analysis *in silico* Evaluation

Different primer pairs targeting the archaeal 16S rRNA gene region have been selected from recent publications (Klindworth et al., 2013; Koskinen et al., 2017). The main criteria for selection were: (a) specificity for archaea *in silico*; (b) low or no amplification of eukaryotic DNA; (c) amplicon length between 150 to 300 bp, suitable for NGS such as Illumina MiSeq. In addition, three “universal” primer pairs (Caporaso et al., 2012; Klindworth et al., 2013; Walters et al., 2016) were tested in parallel to determine their efficiency in detecting archaea in human samples. Full information on the selected primer pairs is given in Table 1.

**TABLE 1:** Primer selection and results of the pre-analysis *in silico* evaluation of all primer pairs used.

**Primer pair**	**Name**	**Primer name^∗^**	**Sequence (5′– >3′)**	**Fragment size (bp)**	**0 mismatch**			**1 mismatch**		
					**Archaea**	**Bacteria**	**Eukarya**	**Archaea**	**Bacteria**	**Eukarya**
1	344F	S-D-Arch-0344-a-S-20	ACGGGGYGCAGCAGGCGCGA	571	46.1%	0.0%	0.0%	67.4%	0.0%	0.0%
	915R	S-D-Arch-0911-a-A-20	GTGCTCCCCCGCCAATTCCT							
2	349F	S-D-Arch-0349-a-S-17	GYGCASCAGKCGMGAAW	566	71.8%	0.0%	0.0%	86.1%	0.0%	0.0%
	915R	S-D-Arch-0911-a-A-20	GTGCTCCCCCGCCAATTCCT							
3	344F	S-D-Arch-0344-a-S-20	ACGGGGYGCAGCAGGCGCGA	697	51.5%	0.0%	0.0%	72.10%	0.0%	0.0%
	1041R	S-D-Arch-1041-a-A-18	GGCCATGCACCWCCTCTC							
4	349F	S-D-Arch-0349-a-S-17	GYGCASCAGKCGMGAAW	692	71.2%	0.0%	0.0%	88.90%	0.0%	0.0%
	1041R	S-D-Arch-1041-a-A-18	GGCCATGCACCWCCTCTC							
5	519F	S-D-Arch-0519-a-S-15	CAGCMGCCGCGGTAA	522	79.3%	0.0%	0.0%	93.4%	0.0%	0.0%
	1041R	S-D-Arch-1041-a-A-18	GGCCATGCACCWCCTCTC							
6	344F	S-D-Arch-0344-a-S-20	ACGGGGYGCAGCAGGCGCGA	462	48.3%	0.0%	0.0%	70.6%	0.0%	0.0%
	806R	S-D-Arch-0786-a-A-20	GGACTACVSGGGTATCTAAT							
7	349F	S-D-Arch-0349-a-S-17	GYGCASCAGKCGMGAAW	457	75.2%	0.0%	0.0%	89.9%	0.0%	0.0%
	806R	S-D-Arch-0786-a-A-20	GGACTACVSGGGTATCTAAT							
8	519F	S-D-Arch-0519-a-S-15	CAGCMGCCGCGGTAA	287	85.6%	6.8%	0.0%	94.9%	90.8	0.1%
	806R	S-D-Arch-0786-a-A-20	GGACTACVSGGGTATCTAAT							
9	349F	S-D-Arch-0349-a-S-17	GYGCASCAGKCGMGAAW	170	79.3%	0.0%	0.0%	91.7%	0.0%	0.1%
	519R	S-D-Arch-0519-a-A-16	TTACCGCGGCKGCTG							
10	519F	S-D-Arch-0519-a-S-15	CAGCMGCCGCGGTAA	266	88.9%	88.8%	0.6%	95.1%	94.9%	1.2%
	**785R**	S-D-Bact-0785-b-A-18	TACNVGGGTATCTAATCC							
11	**515F**	515F-original	GTGCCAGCMGCCGCGGTAA	291	52.9%	86.8%	0.0%	94.3%	93.8%	0.3%
	**806uR**	806R-original	GGACTACHVGGGTWTCTAAT							
12	**515FB**	515F-modified	GTGYCAGCMGCCGCGGTAA	291	85.7%	87.7%	0.0%	95.1%	93.9%	1.4%
	**806RB**	806R-modified	GGACTACNVGGGTWTCTAAT							

*Coverage of *Archaea*, *Bacteria* and *Eukarya* is given in percentages, depending on whether no or one mismatch was allowed (last base at the 3′ end excluded for mismatch). ^∗^According to Klindworth et al. (2013). Designated “universal” primers (primer pairs 10–12) are indicated in bold letters.*

*In silico* evaluation of the selected primer pairs was performed using the online tool TestPrime1.0 (Klindworth et al., 2013) and the non-redundant SILVA database SSU132 (Quast et al., 2013). Two of the primers (344F and 519F) were also tested using TestProbe 3.0 (Klindworth et al., 2013) and the SILVA database SSU132 to assess their individual coverage for the archaeal domain. These two primers were further tested either due to low coverage of the Thaumarchaeota domain (such as primer combinations including the 344F primer) or due to their additional coverage of non-archaeal domains (primer combinations including the 519F).

### PCR and Library Preparation

For archaea-targeting PCR, a nested approach was chosen to increase the specificity for archaea and to avoid the formation of primer dimers caused by the tag attached to the primers, necessary for Illumina sequencing (Peng et al., 2015; Koskinen et al., 2017). Due to the high background DNA from human tissues, the nested approach has proven useful in a variety of samples.

In addition to the nested approach, a standard PCR was performed with three different universal primer pairs (515F-806uR, 515FB-806RB, and 519F-785R), and one archaeal primer pair (519F-806R) for comparative reasons, and to test if a universal approach is capable to cover archaea in human samples in sufficient depth. All primer combinations (in total 27) used for the PCR reactions are provided in Table 2.

**TABLE 2:** displays all primer pair combinations used for the first and the second PCR of the nested approach and the “universal” PCR.

**PCR #**	**Primer combination 1st PCR**	**Primer combination 2nd PCR**
PCR21	349F-915R	Illu 349F-Illu519R
PCR22	349F-915R	Illu 519F-Illu785R
PCR23	349F-915R	Illu 519F-Illu806R
PCR31	344F-1041R	Illu 349F-Illu519R
PCR33	344F-1041R	Illu 519F-Illu785R
PCR34	344F-1041R	Illu 519F-Illu806R
PCR41	349F-1041R	Illu 349F-Illu519R
PCR42	349F-1041R	Illu 519F-Illu785R
PCR43	349F-1041R	Illu 519F-Illu806R
PCR61	349F-806R	Illu 349F-Illu519R
PCR62	349F-806R	Illu 519F-Illu785R
PCR63	349F-806R	Illu 519F-Illu806R
PCR71	519F-1041R	Illu 519F-Illu785R
PCR72	519F-1041R	Illu 519F-Illu806R
PCR81	519F-806R	Illu 519F-Illu785R
PCR82	519F-806R	Illu 519F-Illu806R
PCR91	344F-519R	Illu 349F-Illu519R
PCRQ1	344F-915R (QIAGEN)	Illu 349F-Illu519R
PCRQ3	344F-915R (QIAGEN)	Illu 519F-Illu785R
PCRQ4	344F-915R (QIAGEN)	Illu 519F-Illu806R
PCRM1	344F-915R (NEB Monarch)	Illu 349F-Illu519R
PCRM3	344F-915R (NEB Monarch)	Illu 519F-Illu785R
PCRM4	344F-915R (NEB Monarch)	Illu 519F-Illu806R
PCRA1	344F-915R (Analytik Jena)	Illu 349F-Illu519R
PCRA3	344F-915R (Analytik Jena)	Illu 519F-Illu785R
PCRA4	344F-915R (Analytik Jena)	Illu 519F-Illu806R
PCRQ5	344F-806R (QIAGEN)	Illu 349F-Illu519R
PCRQ6	344F-806R (QIAGEN)	Illu 519F-Illu785R
PCRQ7	344F-806R (QIAGEN)	Illu 519F-Illu806R
PCRM5	344F-806R (NEB Monarch)	Illu 349F-Illu519R
PCRM6	344F-806R (NEB Monarch)	Illu 519F-Illu785R
PCRM7	344F-806R (NEB Monarch)	Illu 519F-Illu806R
PCR8-Uni	n.a.	Illu 515F-Illu806uR
PCR9-Uni	n.a.	Illu 515FB-Illu806RB
PCR10	n.a.	Illu 519F-Illu806R
PCR11-Uni	n.a.	Illu 519F-Illu785R

*If not indicated otherwise (in brackets), the first PCR was followed by a purification of the PCR product by the MinElute PCR Purification kit (QIAGEN) kit. n.a., not applicable.*

For the first PCR, each reaction was performed in a final volume of 20 μl containing: TAKARA Ex Taq^®^ buffer with MgCl_2_ (10 X; Takara Bio Inc., Tokyo, Japan), primers 500 nM, BSA (Roche Lifescience, Basel, Switzerland) 1 mg/ml, dNTP mix 200 μM, TAKARA Ex Taq^®^ Polymerase 0.5 U, water (Lichrosolv^®^; Merck, Darmstadt, Germany), and DNA template (1–50 ng/μl).

After the first PCR, the resulting amplicons were purified to remove primer remnants. This purification was performed with three different kits to compare the different yields and efficiencies, namely MinElute PCR Purification kit (Qiagen; Hilden, Germany), Monarch^®^ PCR & DNA Cleanup Kit (5 μg) (New England Biolabs GmbH; Ipswich, United States), or innuPREP DOUBLEpure Kit (Analytik Jena, Germany) as indicated in Table 2. The purified PCR product was eluted in 10 μl water (Lichrosolv^®^; Merck, Darmstadt, Germany).

From the resulting, purified PCR products, 2 μl were transferred into a subsequent 2nd PCR containing the following mixture: TAKARA Ex Taq^®^ buffer with MgCl_2_ (10 X; Takara Bio Inc., Tokyo, Japan), primers 500 nM, BSA (Roche Lifescience, Basel, Switzerland) 1 mg/ml, dNTP mix 200 μM, TAKARA Ex Taq^®^ Polymerase 0.5 U, and water (Lichrosolv^®^; Merck, Darmstadt, Germany) up to a volume of 25 μL.

The PCR cycling conditions are listed in Table 3, according to the primer pairs used. For all primer pairs, annealing temperatures were either determined experimentally by gradient PCR or adopted from literature.

**TABLE 3:** PCR conditions.

**Target**	**Archaea (16S rRNA gene)**			**“Universal” (16S rRNA gene)**	
**(Nested) PCR, round**	**1°**	**1°**	**2°**	**1°**	**1°**
Primer pair	344F/915R	344F/1041R	All Illumina tagged primer pairs	Illu519F/Illu806R	Illu515F/Illu806uR
	349F/915R	349F/1041R		Illu519F/Illu785R	Illu515FB/Illu806RB
	344F/806R	519F/1041R			
	349F/806R				
	519F/806R				
Initial denaturation	2′, 95°C	5′, 95°C	5′, 95°C	5′, 95°C	3′, 94°C
Denaturation	30″, 96°C (first 10 cycl.), 25″ 94°C	30″, 94°C	40″, 95°C	40″, 95°C	45″, 94°C
Annealing	30″, 60°C	45″, 56°C	2′, 63°C	2′, 63°C	1′, 50°C
Elongation	1′, 72°C	1′, 72°C	1′, 72°C	1′,72°C	1′ 30″, 72°C
Final elongation	10′, 72°C	10′, 72°C	10′, 72°C	10′, 72°C	10′, 72°C
No. of cycles	25	25	30	40	40

*For denaturation, annealing and elongation the corresponding time and temperature is given.*

Sample set 2 was amplified using the primer combination 344F-1041R/519F-806R. For the first PCR, each reaction was performed in a final volume of 20 μl as described above. After the first PCR, the PCR products were purified using Monarch^®^ PCR & DNA Cleanup Kit (5 μg; New England Biolabs GmbH). For the second PCR, the final volume was 25 μl, as described above, only the volume of the DNA template varied: 2 μl purified PCR product for stool and nasal samples, 4 μl for all other samples.

### Next Generation Sequencing, Bioinformatics, and Statistical Analyses

Amplicons were sequenced at the ZMF Core Facility Molecular Biology in Graz, Austria, using the Illumina MiSeq platform (Klymiuk et al., 2016). The MiSeq amplicon sequence data was deposited in the European Nucleotide Archive under the study accession number PRJEB27023.

Data processing for the obtained MiSeq data was performed using the open source package DADA2 (Divisive Amplicon Denoising Algorithm) (Callahan et al., 2016) as described previously (Mora et al., 2016). Shortly, the DADA2 turns paired-end fastq files into merged, denoised, chimera-free, and inferred sample sequences called RSVs. RSVs were classified using the SILVA v128 database as a reference (Quast et al., 2013). In the resulting RSV table, each row corresponds to a non-chimeric inferred sample sequence with a separate taxonomic classification. RSV tables are given in Supplementary Tables S1, S2.

Negative controls (e.g., extraction controls and no-template controls) were included during PCR amplification. The RSVs overlapping the negative controls and samples were either subtracted or completely removed from the data sets. RSVs detected in the negative controls are provided in Supplementary Table S3.

Processing of sequencing data was performed using the in-house Galaxy set-up (Klymiuk et al., 2016) and subsequent statistical analyses were performed in R version 3.4.3 (R Core Team, 2013). Samples were rarefied to 500 reads and alpha diversity was calculated using the Shannon index. Differences between the archaeal diversity indices were tested using Wilcoxon Rank Test. The diversity of the archaeal communities within sample set 2 was determined using two diversity matrices (Shannon and richness). Analysis of variance (ANOVA) was performed to test for differences in the archaeal diversity based on the body location. Principal coordinates analysis (PCoA) based on Bray-Curtis distance was used to visualize differences between the samples from different body site. Redundancy discrimination analysis (RDA) was used to analyze the association between archaeal community composition and the body site location. RDA, alpha diversity and PCoA analysis were performed using Calypso Version 8.62 (Zakrzewski et al., 2016). The RSV tables were used to summarize taxon abundance at different taxonomic levels. The taxonomic profiles obtained at genus level for samples with more than 100 reads were used to generate bar graphs.

A phylogenetic tree was constructed with the obtained archaeal RSVs from sample set 1. The tree-dataset included the RSVs from the universal approaches (515F-806uR, 515FB-806RB, and 519F-785R), the archaeal primer pair 519F-806R, and from the archaeal specific primer pair combination 344F-1041R/519F-806R. The alignment was performed using the SILVA SINA (Pruesse et al., 2012) and the five most closely related available sequences (neighbors) were downloaded together with the aligned sequences. All sequences were cropped to the same length (276 nt, from position 545 nt to 821 nt) and used to construct a tree based on maximum-likelihood algorithm using MEGA7 (Kumar et al., 2016) with a bootstrap value of 500. The Newick output was further processed with iTOL interactive online platform (Letunic and Bork, 2007).

## Results

Primer pairs were evaluated with respect to the following characteristics: high *in silico* specificity for archaeal 16S rRNA genes, amplicon length of 150 to 300 bp (suitable for NGS), and *in vitro* capability to amplify diverse archaeal 16S rRNA genes from a variety of human specimens.

Besides archaea-specific primer pairs, two widely used “universal” primers (Caporaso et al., 2012; Walters et al., 2016), namely 515F-806uR (original) and 515FB-806RB (modified), were evaluated as well to assess the potential of “universal” primers to display archaeal diversity associated with the human body.

### Most Archaea-Targeting Primers Reveal Good Coverage *in silico*

A total of 12 different primer pairs were evaluated *in silico* (Table 1). Most primer pairs showed high coverage for the archaeal domain ranging from 46% to 89% and revealed a high domain-specificity (8 of 12 primer pairs showed no matching outside of the archaeal domain). When one mismatch per primer was allowed, the coverage increased (67% to 95%).

The designated archaeal primer pair 519F-806R was found to target additional sequences within the Bacteria and Eukarya. For instance, when one mismatch per primer was allowed, a >90% *in silico* coverage across the bacterial domain was observed.

We further evaluated the coverage of the primer pairs with respect to specific archaeal groups of particular interest in human archaeome studies, which are the phyla Euryarchaeota, Thaumarchaeota, and Woesearchaeota, as well as the genera *Nitrososphaera, Methanobrevibacter*, *Methanosphaera* and *Methanomassiliicoccus*. For all subsequent *in silico* analyses we allowed one mismatch per primer at all locations except at the last base of the 3′ end.

All primer pairs revealed a high coverage of ≥90% for the Euryarchaeota phylum. For the genus *Methanobrevibacter*, all primer pairs showed a coverage >94%, and for *Methanomassiliicoccus* a coverage >92%. The coverage for *Methanosphaera* was found to be <90% with the exception of primer pairs 519F-806R and 349F-519R, which showed 90% and 90.3% coverage, respectively (Table 4).

**TABLE 4:** *In silico* analysis of the coverage of chosen primer pairs for specific archaeal taxa of interest.

		**Euryarchaeota**				**Thaumarchaeota**		**Nanoarchaeota**
**Primer pair**	**Name**	**Total**	***Methanobrevibacter***	***Methanosphaera***	***Methanomassiliicoccus***	**Total**	***Nitrososphaera***	**Woesearchaeota**
1	344F	89.80%	94.90%	81.00%	100%	20.40%	87.10%	55.80%
	915R							
2	349F	89.70%	95.00%	83.00%	100%	91.2%	89.30%	70.30%
	915R							
3	344F	89.90%	94.30%	78.20%	100%	20.60%	89.00%	56.60%
	1041R							
4	349F	90.20%	94.40%	78.60%	100%	95.80%	92.30%	73.40%
	1041R							
5	519F	94.60%	97.40%	84.60%	92.90%	96.50%	90.60%	82.40%
	1041R							
6	344F	91.50%	95.20%	82.20%	100%	23.20%	88%	55.00%
	806R							
7	349F	91.40%	95.30%	84.20%	100%	96.10%	90.10%	72.60%
	806R							
8	519F	96.30%	98.60%	90%	95%	96.50%	89.50%	82.90%
	806R							
9	349F	91.90%	95.60%	90.30%	95%	97.50%	94.40%	83.10%
	519R							
10	519F	96.20%	98.40%	89.60%	95%	96.00%	86.90%	87.30%
	785R							
11	515F	95.90%	98.30%	89.60%	95%	94.60%	86.90%	89.10%
	806R							
12	515FB	95.90%	98.30%	89.60%	95%	96.40%	89%	89.10%
	806RB							

*One mismatch was allowed per primer (last base at the 3′ end was excluded for mismatch). For primer full names and sequences, please refer to Table 1.*

The coverage of the Woesarchaeota and Thaumarchaeota clades were found to be variable, depending on the primer pairs. While for Woesearchaeota all primer pairs showed coverages between 55% and 89.1%, most analyses that included the primer 344F showed a low *in silico* coverage for Thaumarchaeota (<30%). However, all other primer pair combinations revealed a high coverage of this phylum (>90%; Table 4). The coverage for *Nitrososphaera* in particular varied across primer pairs between 86.9% and 94.4%.

As the archaeal primer 344F has often been used for detecting archaea in a variety of environmental samples (Zhang et al., 2014; Fontana et al., 2016), we further analyzed its coverage using TestProbe 3.0 (Klindworth et al., 2013) and the SILVA database SSU132 (Quast et al., 2013). The results revealed 73.2% coverage of the archaeal domain, a high coverage of the Euryarchaeota phylum (93.8%) and the genera within, especially *Methanobrevibacter* with 96.1% coverage, *Methanosphaera* with 89.9% and *Methanomassiliicoccus* with 100%. The results also revealed a good coverage of 74.6% for Woesearchaeota, but showed, despite a high coverage for the genus *Nitrososphaera* (93.6%), a generally low coverage of the Thaumarchaeota phylum with only 24%. These findings indicate a potentially low capacity of this primer for studies with a focus on thaumarchaeotal diversity.

Additionally, we also analyzed the primer 519F using the TestProbe 3.0, especially since the sequence of the primer 519F (S-D-Arch-0519-a-S-15; 5′-CAGCMGCCGCGGTAA-3′) overlaps with the sequence of the “universal” primer S-^∗^-Univ-0519-a-S-18 (5′- CAGCMGCCGCGGTAATWC-3′). As expected, the results from the *in silico* analysis indicated that the primer 519F targets Bacteria (coverage 98%), Archaea (98.2%) and Eukarya (96.4%). The universal primer S-^∗^-Univ-0519-a-S-18 shows a similar coverage for the three domains: Bacteria (coverage 97.5%), Archaea (96.4%), and Eukarya (95.6%). Considering our *in silico* results, the primer 519F cannot be considered to target Archaea specifically and should be re-named to S-D-Univ-0519-a-S-15.

As most selected archaea-targeting primers analyzed revealed a good coverage of the archaeal domain, all primer pairs were used for subsequent wet-lab experiments.

### Archaeal Community Composition Varies According to the Used Primer Pairs Whereas Universal Primers Fail to Detect the Archaeal Diversity

Herein, we sought to identify the optimal primer pair for amplicon sequencing of the archaeomes in human samples. For this, we selected five representative sample types from different body sites: nose (upper nasal cavity), oral (subgingival sites), stool and appendix specimens, and skin (back) (sample set 1).

Next generation sequencing was performed after a two-step nested PCR (for archaea) or a single-step PCR (“universal” target). The nested PCR approach was selected based on the reasons given in the section “Materials and Methods.” In brief, the first PCR was intended to select the archaeal community and the second to further amplify the archaeal signal.

The results obtained after amplification, NGS, and data analysis based on DADA2 algorithm (Callahan et al., 2016; Koskinen et al., 2017) are summarized in Supplementary Table S4. This table includes the number of total reads obtained and the number of observed ribosomal sequence variants (RSVs) assigned to Bacteria, Archaea and Eukarya (plus unclassified taxa).

The use of universal primers (515F-806uR, 515FB-806RB, and 519F-785R) resulted in reads classified mainly within the bacterial domain, whereas only a small amount of the reads (0-0.3%, stool sample) was classified within the Archaea. The widely used primer pair 515FB-806RB (modified) could not detect a single archaeal RSV, whereas the 515F-806uR (original) resulted in one found RSV in the stool sample (Supplementary Table S4). This confirmed our previous observation that universal primers are mostly not suitable for a detailed study of the archaeome (Koskinen et al., 2017).

The universal primer pair 519F-785R yielded slightly better results, allowing the detection of three different archaeal RSVs from two different samples: *Methanobrevibacter* and *Methanosphaera* in the stool sample and one RSV from the nasal sample, which was classified within the Thaumarchaeota clade. Very similar detection results were obtained using the primer pair 519F-806R. However, this primer pair was originally described to be archaea-specific, but revealed in our study broad *in silico* and experimental coverage of the bacterial and archaeal domain (see previous chapter and Supplementary Table S4).

**FIGURE 1:**
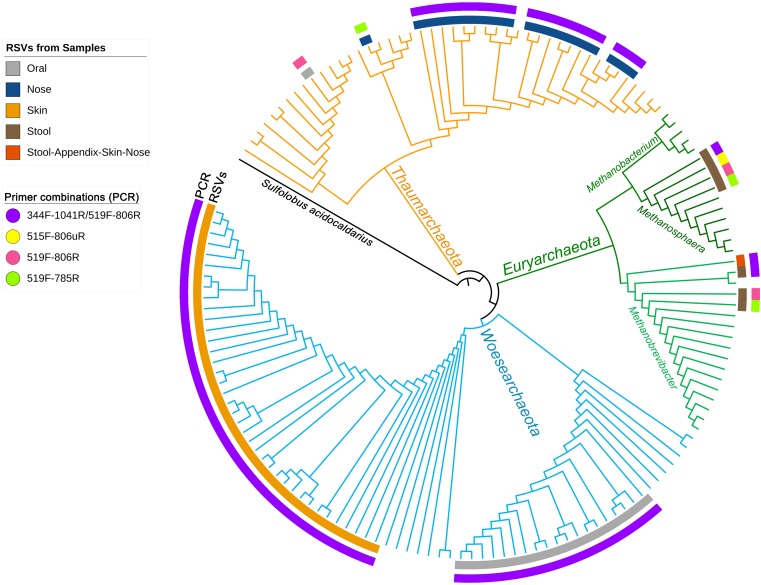
Phylogenetic tree based on the retrieved RSVs from the universal approach with the primer pairs 515F-806uR and 519F-785R, archaeal approach with primer pair 519F-806R and from the PCR based on the primer pair combination 344F-1041R/519F-806R as indicated in colors as an outer circle [legend “Primer combinations (PCR)”]. The inner circle represents the body site from where the RSVs were identified (see legend). Reference sequences from the SILVA database are shown without label/circle. The branches of the tree were colored according to the phyla: blue, Woesearchaeota; green, Euryarchaeota; and orange, Thaumarchaeota.

To visualize the overlap of phylotypes detected by different primer pair approaches, a phylogenetic tree was constructed (Figure 1). Besides the obtained archaeal RSVs from the universal approaches (primer pairs 515F-806uR, 519F-785R, and 519F-806R), the RSVs retrieved from the archaea-specific primer pair combination (344F-1041R/519F-806R) were included for comparison. Overall, 82 individual archaeal RSVs were detected: 20 RSVs in the nasal sample, 19 RSVs in the oral, one RSV in the appendix, 3 RSVs in the stool, and 39 RSVs in the skin sample.

Although the information received from universal and archaea-specific approaches were similar with respect to the detection of methanogenic archaea from stool samples (*Methanobrevibacter* and *Methanosphaera* clade, Figure 1), the universal approaches failed to cover the diversity of Thaumarchaeota (nasal samples) and Woesearchaeota (skin and oral samples).

Ten out of 23 primer pair combinations allowed the detection of archaeal signatures in all analyzed samples (Supplementary Table S4). All 23 primer pair combinations were able to detect archaeal reads in at least one of the sample types analyzed. For example all primer pair combinations detected archaeal RSVs in the stool sample; the number of RSVs, however, varied according to the used primer pair combination.

In the next step, we aimed to identify the optimal primer pair for the detection of archaeal signatures in a variety of human samples. However, depending on the used primer pair, the archaeal community composition was found to be variable (Supplementary Figure S1). We particularly observed that the detected variation in the archaeal composition could be attributed to the primer pair used in the first PCR, whose purpose was to select the communities, while primer pairs used in the second PCR enhanced the signal of the first PCR (Supplementary Figure S1).

It shall be mentioned that for the second PCR only three different primer pairs were used (i.e., 349F-519R, 519F-785R, and the 519F-806R) of which the first two primer pairs had been used before to explore archaeal communities in human samples (Koskinen et al., 2017) and in confined habitats (Mora et al., 2016).

**FIGURE 2:**
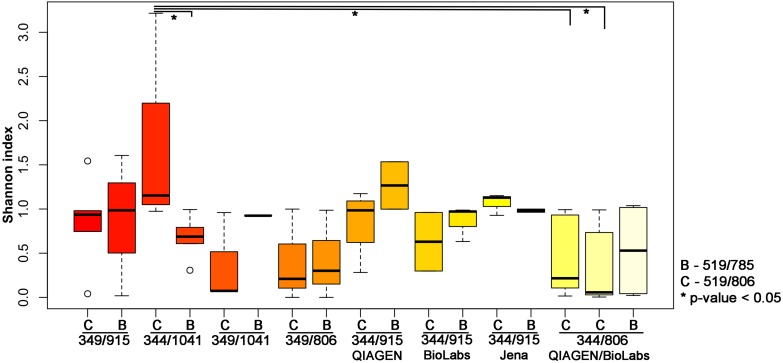
Shannon index indicating the diversity received from different PCR approaches. The results have been plotted and grouped according to the first PCR used and the statistical significance (*p*-value < 0.05; Wilcoxon Rank Test) is indicated by ^∗^.

To further explore the influence of the primer pair selection on the archaeal community composition, the alpha diversity was calculated using the Shannon index (Figure 2). For this analysis, we excluded the results obtained from the second primer pair 349F-519R as most samples yielded less than 500 reads, with the exception of the stool sample.

The highest archaeal diversity was detected with primer combination 344F-1041R/519F-806R (PCR34). This result was found to be significant (*p* < 0.05) compared to PCR 33 (344F-1041R/519F-785R), PCR Q7 (344F-806R/519F-806R) and PCR M7 (344F-806R/519F-806R; Table 2 and Figure 2).

Based on the results from the comparison of the alpha diversities and the lower number of bacterial and eukaryal background signals (Supplementary Table S4), we selected the nested approach with the primer pair 344F-1041R in the first PCR, followed by a second PCR with the primers 519F-806R for subsequent analyses (see below).

It shall be noted that the use of the different purification kits between the first and the second PCR resulted in no significant differences based on the alpha diversity (Shannon index, Wilcoxon Rank Test; Figure 2). However, as the Monarch^®^ PCR & DNA Cleanup Kit (5 μg) (New England Biolabs GmbH; Ipswich, United States) revealed visible band on the gel electrophoresis for the amplicons after the purification, we decided to further use this kit for the purification step.

### The Selected Amplification Approach Revealed Broad Archaeal Diversity in Human Stool, Appendix, Nasal, Oral, and Skin Samples

In a next step, we applied the amplification approach based on the primer pair combination 344F-1041R/519F-806R to a number of additional samples from the following body sites: nasal cavity (*n* = 5), oral cavity (*n* = 6), appendix (*n* = 5), stool (*n* = 5), and skin (*n* = 7) (sample set 2).

**FIGURE 3:**
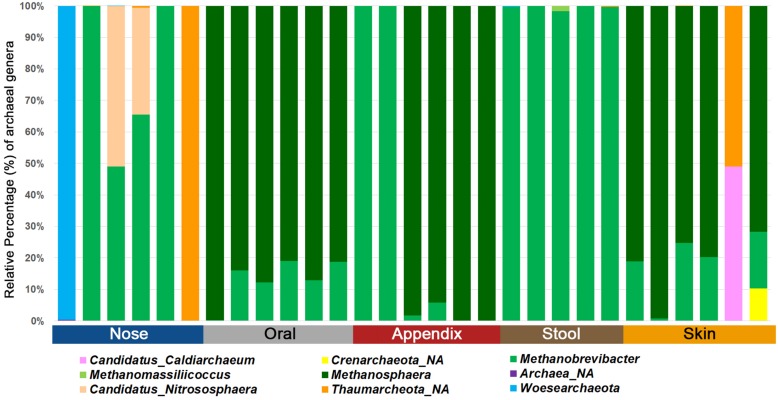
Bar chart displaying the different archaeal lineages detected in different human samples using the superiorly performing primer combination 344F-1041R/519F-806R.

Our selected PCR approach allowed the detection of archaea in all samples investigated. We obtained an average of 102,366 reads and eight observed RSVs for the nasal cavity, 56,480 reads and 35 observed RSVs for the oral samples, 46,022 reads and eight observed RSVs for the appendix samples, 93,948 reads and four observed RSVs for the stool samples, and 76,001 reads and 30 observed RSVs for the skin samples. A summary of the number of archaeal, bacterial and eukaryotic reads/RSVs can be found in Supplementary Table S5. The results on genus level are visualized in Figure 3.

**FIGURE 4:**
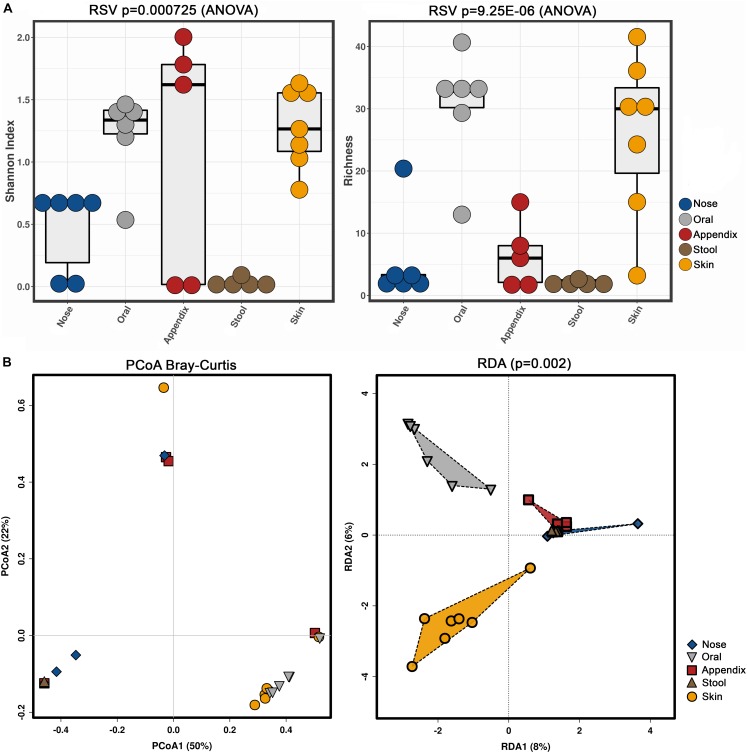
Alpha (**A**, Shannon index and richness) and beta diversity (**B**, PCoA and RDA) analyses of the obtained archaeal community information, based on primer combination 344F-1041R/519F-806R.

Our results confirmed previous findings that archaeal communities are body-site specific (Koskinen et al., 2017), as alpha and beta diversity indices revealed significant differences (Shannon index, richness, RDA plot) (Figure 4).

Notably, the stool samples revealed the overall lowest diversity of archaea, with only three to five identified archaeal RSVs, while skin and oral samples contained a higher diversity, with five to 49 RSVs found in the skin samples and 14 to 49 RSVs in the oral samples.

## Discussion

More than 40 years after the description of archaea as a separate domain of life, knowledge on the composition and function of the human archaeome is still sparse. For instance, it is unclear how humans acquire these, mostly oxygen-sensitive, microorganisms after birth, although it has been reported that archaea can be detected in the human microbiome already in the first year of life (Palmer et al., 2007; Wampach et al., 2017). Additionally, it still remains largely unexplored, how archaeal communities interact/communicate with other human commensal microorganisms. It is unknown, whether archaeal communities are affected by dysbiosis or human disease, or, *vice versa*, are involved in pathogenic situations. Facing these numerous unsolved mysteries, we argue that more studies on the human archaeome are needed.

Although purely PCR-based studies are not capable to distinguish living or dead cells, amplicon-based NGS sequencing remains a method of choice in microbiome research. This procedure allows a reliable, qualitative detection of microorganisms, also in samples with high background DNA.

To address the need for an archaea-specific, amplicon-based NGS protocol for human microbiome samples, we herein tested 12 different primers previously described in literature (Klindworth et al., 2013), in 27 primer pair combinations and evaluated their performance using *in silico* and experimental approaches on five different human sample types.

Despite their overall good *in silico* performance, the three “universal” primer pairs tested in this study failed to picture the archaeal diversity in the wet-lab experiments. Two of them represent the most-used universal primers for amplicon-based microbiome analyses (Caporaso et al., 2012; Walters et al., 2016), but resulted in the detection of only one (515F-806uR) or zero archaeal RSVs (515FB-806RB) in five sample types that evidentially possessed a variety of archaeal signatures.

The reasons for the failure of the universal primers to detect archaeal signatures are unclear. It appears that, depending on the diversity within the sample, bacterial signatures are preferred by slightly superior primer specificity and/or annealing.

Based on the outcome of the tested universal primer pairs, we decided to focus on archaea-specific approaches, with combinations of nine different primer combinations for the first PCR (all archaea-specific), and two archaea-specific and one universal primer pair for the second PCR, resulting in 23 different approaches (Table 2).

We observed that archaeal primer pair 519F-806R, which has been used before for archaea-targeted amplicon sequencing (Siles et al., 2018), detected only a small proportion of the archaeal diversity in the analyzed samples. The result was improved when a nested PCR was performed, with an amplification based on primer pair 344F-1041R in the first PCR.

Nested PCR has been shown to improve sensitivity and specificity and is useful for suboptimal DNA samples (Bomberg et al., 2003; Vissers et al., 2009). Based on our experience in the past (Koskinen et al., 2017), other reports (De Vrieze et al., 2018), and due to the fact that all attempts to use Illumina-tagged archaeal primers to directly identify archaeal 16S rRNA genes in human samples failed, we retained this approach for a qualitative archaeal diversity assessment.

Notably, although the primer pair combinations 344F-915R/349F-519R and 344F-915R/519F-785R had been used earlier to detect archaeal signatures in human and environmental samples (Mora et al., 2016; Koskinen et al., 2017), our study revealed that when the second PCR contained the Illumina-tagged primers 349F-519R, almost no reads in samples other than the stool were retrieved (Supplementary Table S4).

Ten out of the 23 different primer combinations allowed the detection of archaeal signatures in all analyzed samples (sample set 1). The results of two of the primer pair combinations were outstanding regarding the number of reads and observed RSVs in each sample (Supplementary Table S4), namely combinations 344F-1041R/519F-806R and 344F-1041R/519F-785R. The comparison of the retrieved alpha diversity (based on Shannon index) indicated that the archaeal diversity uncovered with the primer pair 344F-1041R/519F-806R was significantly higher than the one obtained with the primer pair combination 344F-1041R/519F-785R (Figure 2).

According to the obtained results, we decided to use the primer pair combination 344F-1041R/519F-806R to identify and characterize archaeal communities within human samples. Despite the fact that the second primer pair 519F-806R performs like a universal primer pair and also reads classified within Bacteria and Eukarya were retrieved along with the archaeal reads, this procedure still proved to be superior for the detection of the archaeal diversity.

To further test and validate this primer pair, we analyzed 29 additional samples from different body sites (nasal cavity, oral, appendix, stool, skin; sample set 2), resulting in the detection of overall 85 archaeal RSVs classified within six different phyla.

Based on these data, we were able to confirm body-site specificity of the human archaeome (Koskinen et al., 2017). In detail, the gastrointestinal tract (stool and appendix samples) and the oral samples were found to be predominated by distinctive euryarchaeal signatures, whereas the nasal cavity was predominated by a mixture of Euryarchaeota and Thaumarchaeota signatures. The skin revealed a mixture of Euryarchaeota, Thaumarchaeota, Aenigmarchaeota, and, in very low amounts also Crenarchaeota, confirming previous results (Tsai et al., 2016; Koskinen et al., 2017; Moissl-Eichinger et al., 2017) and the suitability of the selected primer-pair combination to picture the local archaeome in a variety of samples.

## Conclusion

We have shown that the choice of the primers influences substantially the picture of the archaeal community in amplicon-based microbiome studies. Our results have indicated the importance of archaea-specific procedures, as universal approaches fail to picture the diversity of archaeal signatures. In our survey, a nested PCR approach based on primer pair 344f-1041R for the first PCR, followed by a second PCR with the primer pair 519F-806R was found to be superior for the analysis of the archaeome of gastrointestinal tract, oral cavity, and skin. This protocol for archaeal signature detection might also be useful for samples from other environments and holobionts, such plants or animals.

## Data Availability Statement

The MiSeq amplicon sequence data was deposited in the European Nucleotide Archive under the study accession number PRJEB27023.

## Ethics Statement

Research involving human material was performed in accordance with the Declaration of Helsinki and was approved by the local ethics committees (the Ethics Committee at the Medical University of Graz, Graz, Austria). Bacterial microbiome studies of some of the samples used in this study have already been published elsewhere [oral, nasal, skin samples (Klymiuk et al., 2016; Santigli et al., 2016; Koskinen et al., 2018)]. Details of the ethics approvals obtained are shown there. Appendix samples and stool samples have been obtained covered by the ethics votes: 25-469 ex12/13 and 27-151 ex 14/15.

## Author Contributions

MP and CM-E designed the study. GS, HT, VS, ES, BK, and CH provided the clinical samples. CC and MP prepared the 16S rRNA gene amplicons for sequencing. MP contributed to the bioinformatics and statistical analysis, visualization, and interpretation of the data. MB provided the discussion, technical support, and corrected the manuscript. All authors read, corrected, and approved the final manuscript.

## Conflict of Interest

The authors declare that the research was conducted in the absence of any commercial or financial relationships that could be construed as a potential conflict of interest.
