# Supplementary material for: Exploring the Archaeome: Detection of Archaeal Signatures in the Human Body
Source: Front Microbiol. 2019 Dec 5;10:2796. doi: 10.3389/fmicb.2019.02796 (PMC6906140; doi:10.3389/fmicb.2019.02796)
Supplement: Supplementary file 1 [file Image_1.pdf]

Supplementary Fig. 1

Exploring the archaeome: detection of archaeal signatures in the human body

Manuela R. Pausan[1], Cintia Csorba[1,+], Georg Singer[2], Holger Till[2], Veronika Schöpf[3,5], Elisabeth Santigli[4], Barbara Klug[4], Christoph Högenauer[1], Marcus Blohs[1], Christine Moissl-Eichinger[1,5,*]

[1] Department of Internal Medicine, Medical University of Graz, Graz, Austria

[2] Department of Pediatrics and Adolescent Surgery, Medical University of Graz, Graz, Austria

[3] Institute of Psychology, University of Graz, Graz, Austria

[4] Department of Dental Medicine and Oral Health, Medical University Graz, Graz, Austria

[5] BioTechMed, Graz, Austria

[+] Present address: AIT Austrian Institute of Technology GmbH, Tulln, Austria

[*] Corresponding author

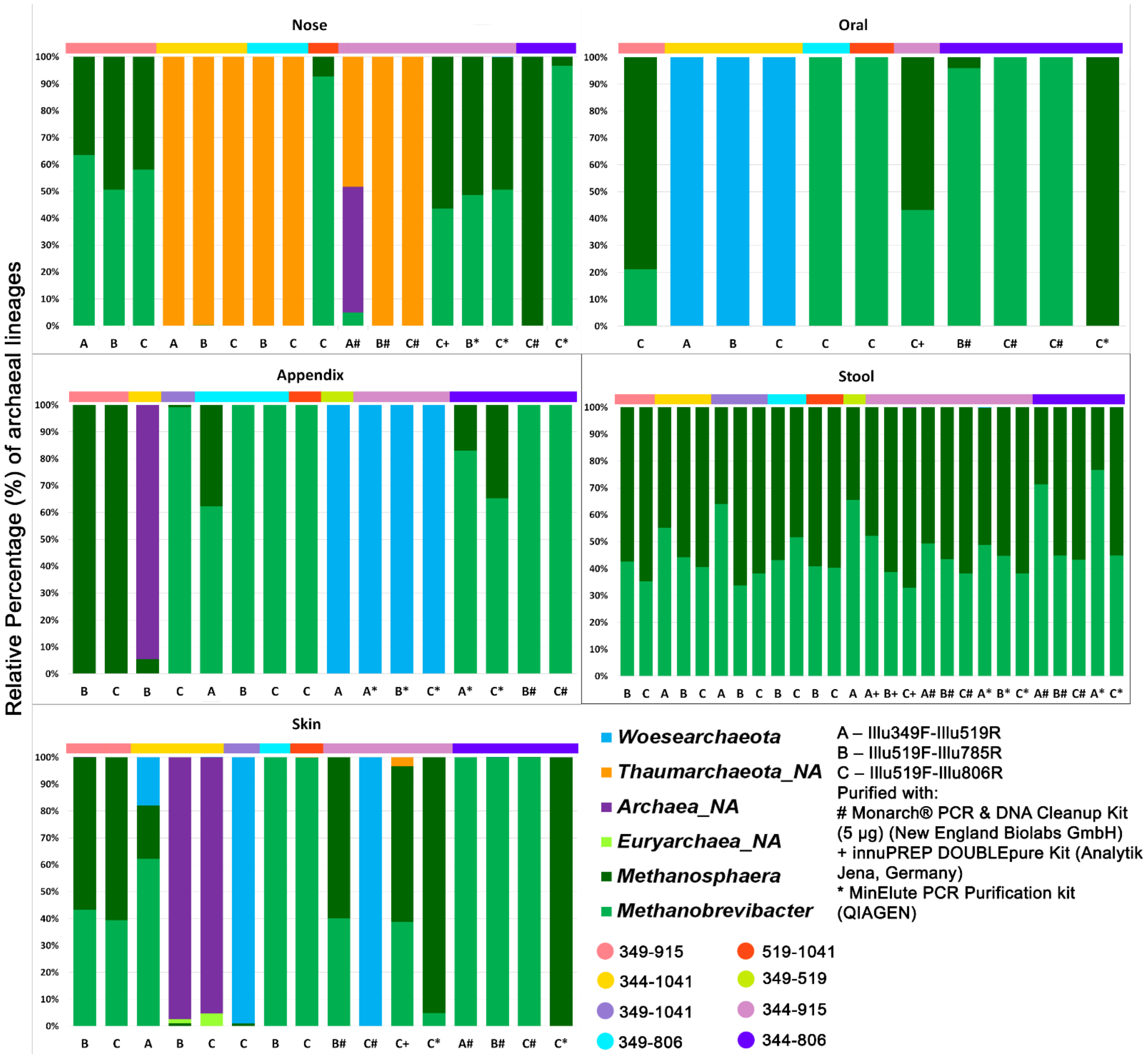

Suppl. Fig. 1: Bar charts illustrating the diversity of archaeal communities on genus level in dependence of primer pair combinations. Primer combinations are illustrated via the colored bars above the columns (first primer pair), and by letters below the columns (second primer pair). In addition, information is given on the different purification kits that have been used between the two steps of the nested PCR. If no character is given next to the letter, MinElute PCR Purification kit (QIAGEN) has been used to purify the PCR product. Only samples with more than 100 archaeal reads have been used for creating the bar charts.
